# Comparative efficacy of a multi-epitope DNA vaccine via intranasal, peroral, and intramuscular delivery against lethal *Toxoplasma gondii* infection in mice

**DOI:** 10.1186/1756-3305-7-145

**Published:** 2014-03-31

**Authors:** Hua Cong, Quan Yuan, Qunli Zhao, Lingxiao Zhao, Huiquan Yin, Huaiyu Zhou, Shenyi He, Zhiyu Wang

**Affiliations:** 1 Department of human parasitology, Medical school, Shandong University, No.44 Wenhuaxi Road, Jinan, Shandong 250012, P. R. China; 2 School of Mechanical Engineering, Shandong University, Jinan, Shandong 250061, P. R. China; 3 School of Public Health, Shandong University, No.44 Wenhuaxi Road, Jinan, Shandong 250012, P. R. China

**Keywords:** *Toxoplasma gondii*, Multi-epitopes, *Salmonella typhimurium*, Vaccine

## Abstract

**Background:**

*Toxoplasmosis* is an important zoonosis, being a cause of congenital disease and abortion in animals and humans. DNA vaccination as a promising vaccine remains a challenge for an improved delivery system.

**Methods:**

In this study, attenuated *Salmonella typhimurium* BRD509 was used to deliver a DNA vaccine encoding several epitopes, derived from the tachyzoite proteins SAG1, GRA1, ROP2, GRA4 and bradyzoite proteins SAG2C, SAG2X of *Toxoplasma gondii* and A_2_/B subunit of *cholera toxin*. The recombinant plasmids were electroporated into attenuated *Salmonella typhimurium*. Humoral and cellular immune responses were evaluated for BALB/c mice administered with this attenuated recombinant *Salmonella* vaccine via the oral and nasal route or by intramuscular injection with DNA plasmid directly.

**Results:**

High IgG levels were present in the mice immunized intramuscularly, while IgA levels were higher in the oral and nasal immunization groups. Furthermore, cellular immunity was activated in oral immunization groups with 60% survival rate following challenge with high virulent RH strain.

**Conclusions:**

The results from this study indicate that a DNA vaccine encoding multi-epitopes of *T. gondii* delivered by attenuated *Salmonella* is promising.

## Background

*Toxoplasma gondii* is a single-cell obligate intracellular protozoan, which is widely prevalent in humans and animals [1,2]. This parasite is of major medical and veterinary importance, being a cause of congenital disease and abortion [3,4]. Despite many efforts and significant advances in the understanding of the immune responses that occur after infection by *T. gondii*, animal experiments have shown that the effect of monovalent antigen vaccine is not ideal [5-7]. Therefore, the development of a variety of antigen combinations for different stages of life cycle, especially epitopes from tachyzoites and bradyzoites are likely to be needed for full protection [8-11]. We constructed a DNA vaccine encoding five epitopes of *T. gondii* previously. The vaccination injected intramuscularly into BALB/c resulted in an improvement of the humoral and cellular immune response in immunized mice, but there was only 20% survival rate achieved in vaccinated mice [12]. In order to broaden the immunogenicity, we intend to include more epitopes from various proteins of tachyzoites and bradyzoites in the vaccine construction. Other than epitopes from tachyzoite proteins SAG1, GRA1, ROP2, GRA4, epitopes from bradyzoite proteins SAG2C, SAG2X were also included [13,14].

However, DNA vaccines have been used to inject intramuscularly as naked DNA using a gene gun and by subcutaneous inoculation. After vaccination, only a limited amount of DNA reaches professional APCs cells [15]. Mechanisms to induce a more effective immune response and to improve protection from immunized mice remains to be determined. Recombinant *Salmonella* have been used as vaccine vectors to deliver both DNA and protein vaccines from a wide variety of bacterial, viral and parasitic sources [16,17]. Attenuation virulence bacteria, which can penetrate host cells delivering vaccine antigen to APCs, can be used effectively to transport immunogens [18,19].

In this study, a recombinant attenuated *Salmonella* DNA vaccine encoding multi-epitopes of *T. gondii* and A_2_/B subunits of *cholera toxin* was constructed. The immunity induced by this attenuated recombinant *Salmonella* vaccine administered orally and nasally in BALB/c mice was compared to immunity induced by a plasmid DNA vaccine injected into mice intramuscularly. Protection against challenge with high virulent RH strain of *T. gondii* was evaluated.

## Methods

### Parasites and bacterial strain

Tachyzoites of the high virulent RH strain of *T. gondii* were cryopreserved in our laboratory. Parasites were maintained by serial intraperitoneal passage in BALB/c mice. Tachyzoites were harvested from the peritoneal fluid of mice after 72 h, and used to challenge immunized mice.

*Salmonella typhimurium* strain BRD509 is an aroA^-^ and aroD^-^ mutant of SL 1344 [20]. This was kindly provided by Dr. Jifeng Bian, the Department of Molecular Biology, Shandong University.

### Construction of recombinant *Salmonella*

**Figure 1:**
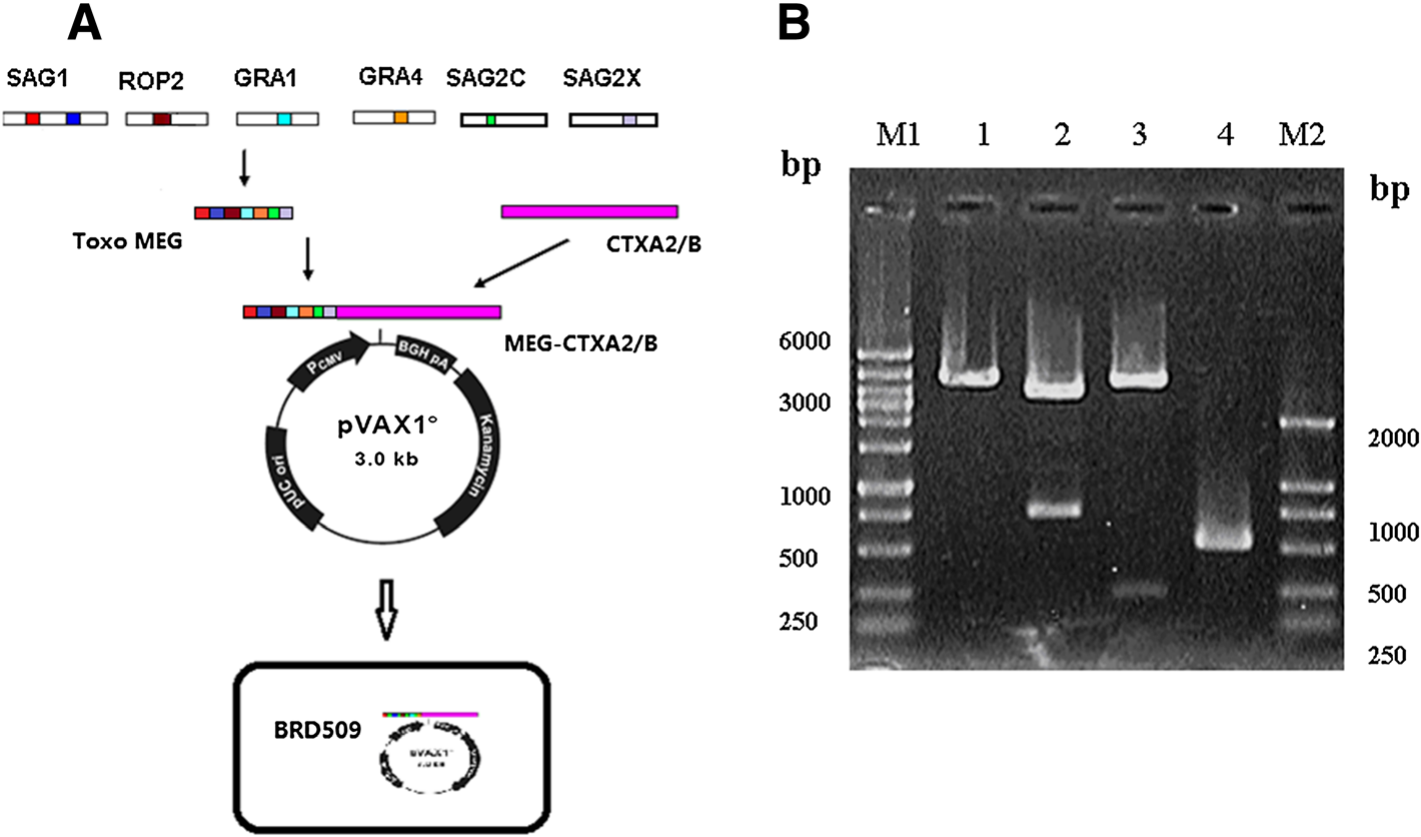
**Construction of recombinant attenuated *Salmonella *carrying pVAX1-MEG-CTXA**_**2**_**/B plasmid encoding multi-epitope genes derived from *T. gondii *and A**_**2**_**/B subunits of *cholera toxin. *(A)** The diagram of the construction of recombinant attenuated *Salmonella.***(B)** Gel electrophoresis analysis of pVAX1-MEG-CTXA_2_/B plasmid encoding multi-epitope genes of *Toxoplasma gondii*. Lane M1: DNA Marker D, L 100 + 6000, Lane M2: DNA Marker DL2000, Lane1: pVAX1-MEG-CTXA_2_/B plasmid digested by EcoR I, Lane 2: pVAX1-MEG-CTXA_2_/B plasmid digested by Hind III and EcoR I, Lane 3: pVAX1-MEG-CTXA_2_/B digested by Hind III and Kpn I, Lane4: PCR product of CTXA_2_/B.

The oligonucleotides of multi-epitope genes (MEG) were designed based on the *T. gondii* peptide sequences: SAG1-I 59–67 (TCPDKKSTA), SAG1-II 246–255 (ILPKLTENPW), GRA1 176–186(DTMKSMQRDED), ROP2 200–215 (GDVVIEELFNRIPETS), GRA4 235–243 (SGLTGVKDS), SAG2C 36–44 (SQFLSLSLL), SAG2X 215–223 (AAGTTATAV). The construction of pVAX1-MEG-CTXA_2_/B was shown in Figure 1.

Attenuated recombinant *Salmonella* carrying pVAX1-MEG-CTXA_2_/B was generated by electroporation. pVAX1-MEG-CTXA_2_/B plasmid was transferred into 100 μl competent *S. typhimurium* BRD509 in the condition of 2.5 kV, 25 uF, 5 ms. To produce inocula, recombinant *Salmonella* were incubated overnight at 37°C until an OD_600_ of 0.8 was reached, then washed and resuspended in PBS to a final density of approximately 1–5 × 10^9^ cfu.

### Vaccinations and challenges

SPF BALB/c female mice (6–8 weeks old) were used in the immunization and parasite challenge. They were purchased from The Laboratory Animal Center of Shandong University. All studies were conducted with Animal Care and Use Committee of Shandong University approval.

The mice were randomly divided into intraoral, intranasal and intramuscular immunization groups (30 mice per group). Table 1 summarizes the treatments performed in the mice. Mice were vaccinated three times at 2 weeks intervals. Blood was collected by orbital plexus puncture and sera were stored at -70 °C for further analysis.

**Table 1:** Summary of treatments performed in the BALB/c mice

**Groups**	**Route of administration**	**Treatments**^**a**^	**Mice number**		
			**Total mice**	**In HI**^**b **^**and CMI**^**c**^	**In challenge**^**d**^
I	Intramuscular vaccination	100 μl PBS	10	3	7
		100 μg pVAX1	10	3	7
		100 μg pVAX1-MEG-CTXA_2_/B	10	3	7
II	Intranasal vaccination	200 μl BRD509	10	3	7
		200 μl BRD509/pVAX1	10	3	7
		200 μl BRD509/pVAX1-MEG-CTXA_2_/B	10	3	7
III	Intraoral vaccination	50 μl BRD509	10	3	7
		50 μl BRD509/pVAX1	10	3	7
		50 μl BRD509/pVAX1-MEG-CTXA_2_/B	10	3	7

^a^The mice were randomly divided into intramuscular, intranasal and intraoral immunization groups (30 mice per group). For the intramuscular group, the mice were injected 100 μl 1 μg/μl recombinant plasmid or empty plasmid in the quadriceps muscle; For the nasal group, each mouse was given a nasal drip of 50 μl 10^9^ cfu/ml *Salmonella* bacteria with recombinant plasmid or empty plasmid; The oral group was administrated 200 μl 10^9^ cfu / ml *Salmonella* bacteria with recombinant plasmid or empty plasmid orally; Each group also had 10 mice which were treated with *Salmonella* without plasmid or saline as negative control. Mice were vaccinated three times at 2 week intervals.

^b^Humoral immunity (HI) was tested by collecting sera from three immunized mice in each group.

^c^Cellular immunity (CMI) was evaluated by splenocytes from three immunized mice per group 2 weeks after the last immunization.

^d^Four weeks after the last immunization, immunized mice were challenged intraperitoneally with 1 × 10^3^ tachyzoites of *T. gondii* RH strain.

For challenge study, immunized mice were challenged intraperitoneally with 1 × 10^3^ tachyzoites of RH strain *T. gondii* 4 weeks after the last immunization. The survival time and the survival rate was measured.

### Measurement of humoral antibodies response

To measure *T. gondii*-specific total IgG and IgA, plates (Dursley, UK) were coated with 10 ug/ml solution of STAg at 4°C overnight. Sera were diluted in 1% PBST-20 (1:100) and incubated in the plates for 1 h at room temperature (RT). After washing the plates, Horseradish peroxidase (HRP)-conjugated goat anti-mouse IgG, IgA (SouthernBiotech, USA) were further incubated for 1 h at RT. Peroxidase activity was revealed by 3,3′,5,5′- tetramethylbenzidine (TMB, 10 mg/ml) and stopped by adding 50 μl of 2 M H_2_SO_4_. The optical density (OD) was read out at 450 nm in a microplate reader (Bio-TEK, USA).

### Proliferation assays

Spleens from three immunized mice per group were removed 2 weeks after the last immunization. The splenocytes were adjusted to a concentration of 5 × 10^6^ cells/ml in DMEM with 10% FCS. The suspension of 100 μl in each well was cultured with STAg (10 μg/ml), purified rMEG protein (50 μg/ml) or concanavalin A (Con A, 5 μg/ml; Sigma). Cell proliferative activity was measured according to instructions described in a previous study [21].

### T lymphocyte subsets analysis

Cells were stained with FITC-labeled anti-mouse CD8^+^ monoclonal antibody and PE-labeled anti-mouse CD4^+^ monoclonal antibody, T lymphocyte subsets were measured using flow cytometry (Beckman Coulter, USA).

### Cytokine assays

Splenocytes from immunized mice were cultured with rMEG protein as described for lymphocyte proliferation assays. Commercial ELISA kits (mouse IL-4 OptEIA, IL-5 OptEIA, IFN-γ OptEIA, IL-2 OptEIA, Endogen, USA) were used according to the manufacturer’s instructions to assay cytokine levels in culture supernatants obtained at 24 h for IL-4, at 36 h for IL-5, at 72 h for IL-2 and at 96 h for IFN-γ.

### Statistical analysis

The statistically significant differences between groups were calculated with one-factor analysis of variance (ANOVA). Differences were considered to be significant with p < 0.05.

## Results

### Construction of recombinant *Salmonella*

The diagram for multi-epitope DNA vaccine of *T. gondii* is shown on Figure 1A. Recombinant *Salmonella* carrying pVAX1-MEG-CTXA_2_/B plasmid were identified by enzyme digestion, by PCR (Figure 1B) and further confirmed by sequencing.

### Humoral immune response

**Figure 2:**
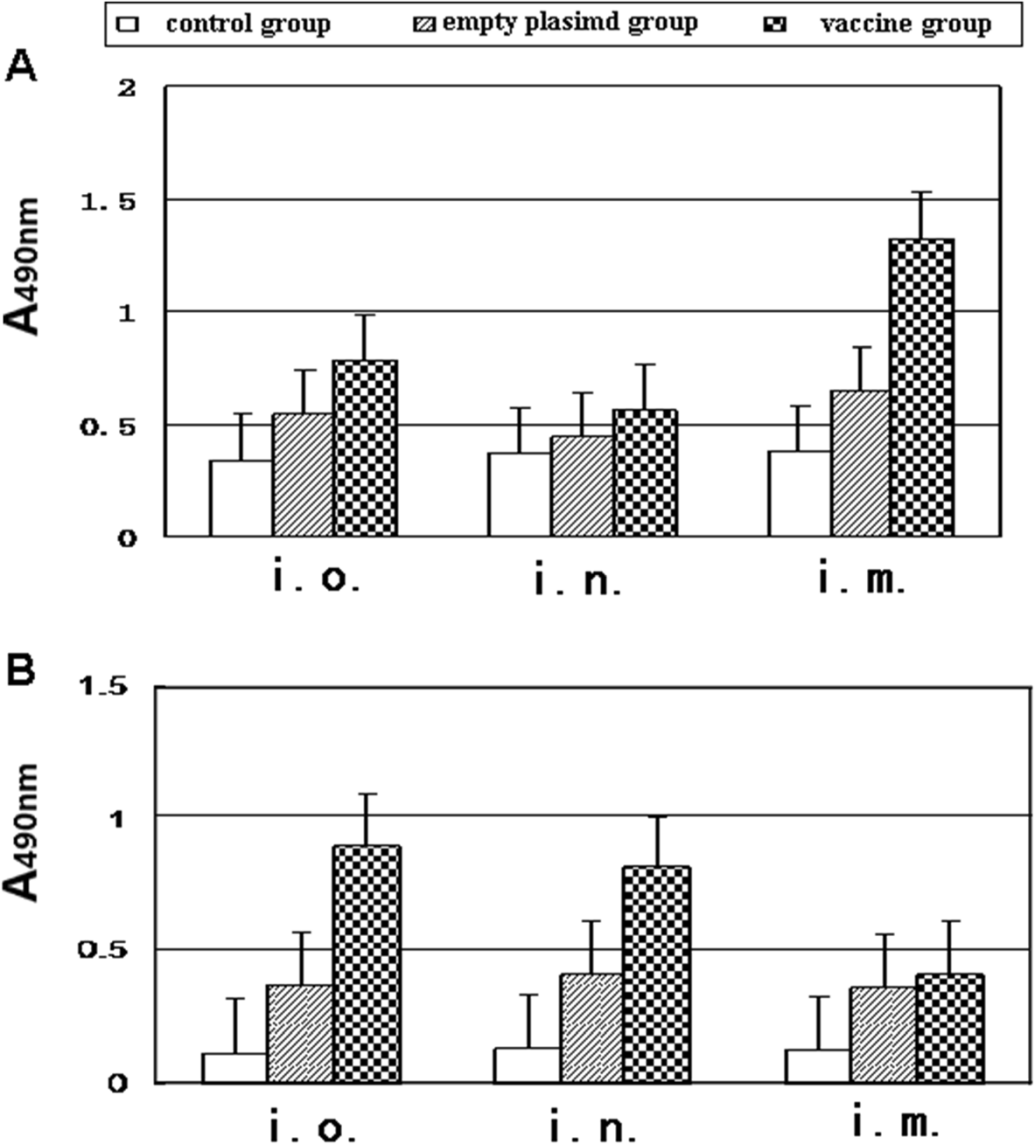
**Specific humoral response to native *T. gondii *antigens in mice immunized with multi-epitope DNA vaccine was measured using ELISA 2 weeks after the last immunization. (A)** Total Anti-*T. gondii* IgG; **(B)** Anti-*T. gondii* IgA were tested by sera diluted 1:200 from BALB/c mice via three different immunized routes: i.o.( intraoral); i.n. (intranasal); i.m. (intramuscular).

IgG antibody levels in mouse serum indicates that pVAX1-MEG-CTXA_2_/B DNA plasmid inoculated intramuscularly induced higher levels of IgG than mice immunized with BRD509/pVAX1-MEG-CTXA_2_/B orally and intranasally, p < 0.05 (Figure 2A). On the other hand, higher levels of anti-*T. gondii* IgA were detected in the serum of mice immunized with BRD509/pVAX1-MEG-CTXA_2_/B via the oral and nasal route than in the serum of mice injected intramuscularly with pVAX1-MEG-CTXA_2_/B plasmid (p < 0.05) (Figure 2B).

### Cellular immune response analysis

CD4^+^ and CD8^+^ T lymphocyte subsets in immunized mice were assayed by flow cytometry. Table 2 shows the percentage of CD4^+^ and CD8^+^ T-cell. The percentage of CD8^+^ T cells in mice immunized intramuscularly with pVAX1-MEG-CTXA_2_/B plasmid was 28.54 ± 0.92%. A even higher percentage of CD8^+^ T cells, 30.01 ± 1.78%, was achieved in mice immunized intranasally with BRD509/ pVAX1-MEG-CTXA_2_/B. The highest percentage of CD8^+^ T-cell, 35.55 ± 0.70%, was achieved in mice immunized orally with BRD509/pVAX1-MEG-CTXA_2_/B (Table 2). Culture supernatants from antigen-stimulated splenocytes from mice in these groups were quantified with sandwich ELISA for Th1 and Th2 cytokines. As shown in Table 3, culture supernatant cytokines from the mice vaccinated intramuscularly with pVAX1-MEG- CTXA_2_/B demonstrated preferential production of IFN-γ and IL-2, which were significantly higher than that of control groups (p < 0.05). The amounts of IFN-γ and IL-2 were significantly higher in the mice via oral and intranasal immunization with recombinant *Salmonella* BRD509 than the mice immunized intramuscularly (p = 0.02). However, the levels of IL-4 and IL-5 produced by immunized mice were low, and no statistically significant differences were present between vaccinated groups and control groups (p > 0.05). Furthermore, antigen specific lymphocyte proliferation activity was highly enhanced for BRD509/pVAX1-MEG-CTXA_2_/B oral and intranasal immunization groups than for the pVAX1-MEG-CTXA_2_/B intramuscular immunization group (p < 0.05).

**Table 2:** **CD4**^**+**^_**, **_**CD8**^**+ **^**subtypes of T cells from immunized mice were measured using flow cytometry**

**Immunization route**^**a**^	**Immunization regimen**	**CD4**^**+**^**(%)**^**b**^	**CD8**^**+**^**(%)**^**b**^
Intramuscular vaccination	PBS	26.71 ± 1.32	21.67 ± 0.82
	pVAX1	25.45 ± 1.13	22.37 ± 0.98
	pVAX1-MEG-CTXA_2_/B	24.56 ± 1.25	28.54 ± 0.92
Intranasal vaccination	BRD509	25.71 ± 0.64	22.37 ± 0.83
	BRD509/pVAX1	24.32 ± 1.75	26.34 ± 0.93
	BRD509/pVAX1-MEG-CTXA_2_/B	24.68 ± 1.43	30.01 ± 1.78
Intraoral vaccination	BRD509	27.45 ± 1.91	24.38 ± 0.98
	BRD509/pVAX1	24.32 ± 1.21	30.28 ± 0.53
	BRD509/pVAX1-MEG-CTXA_2_/B	25.15 ± 1.55	35.55 ± 0.70

^a^Mice were immunized by three immunization routes, intramuscular, intraoral, intranasal, on day 0 and day 14 and day 28 with different immunization regimens.

^b^The splenocyte culture supernatants taken from mice (n = 3, each group) 2 weeks after the last immunization were stained with FITC-labeled anti-mouse CD8^+^ monoclonal antibody and PE-labeled anti-mouse CD4^+^ monoclonal antibody, T lymphocyte subsets were measured using flow cytometry.

**Table 3:** Cell proliferative assay and cytokine production in the splenocyte cultures obtained from immunized mice

**Immunization route**^**a**^	**Immunization regimen**	**Cytokine production (pg/ml)**^**b**^				
		**IL-2**	**IFN-γ**	**IL-4**	**IL-5**	**SI**^**c**^
Intramuscular vaccination	Saline	12 ± 3	13 ± 8	<10	<10	0.23
	pVAX1	16 ± 4	15 ± 5	11 ± 8	<10	0.51
	pVAX1-MEG-CTXA_2_/B	93 ± 21	385 ± 64	15 ± 7	13 ± 6	1.61
Intranasal vaccination	BRD509	13 ± 4	14 ± 5	<10	17 ± 5	0.75
	BRD509/pVAX1	14 ± 7	15 ± 6	<10	19 ± 5	1.07
	BRD509/ pVAX1-MEG-CTXA_2_/B	105 ± 30	412 ± 24	23 ± 6	<10	2.12
Intraoral vaccination	BRD509	12 ± 5	15 ± 4	<10	16 ± 8	0.75
	BRD509/pVAX1	15 ± 7	18 ± 4	<10	16 ± 7	1.45
	BRD509/ pVAX1-MEG-CTXA_2_/B	121 ± 36	507 ± 16	<10	12 ± 4	2.85

^a^Mice were immunized by three immunization routes, intramuscular, intraoral, intranasal on day 0 and day 14 and day 28 with different immunization regimens.

^b^The splenocyte culture supernatants taken from mice (n = 3, each group) 2 weeks after the last immunization were examined for cytokine production by sandwich ELISA obtained at 24 h for IL-4, at 36 h for IL-5, at 72 h for IL-2 and 96 h for IFN-γ.

^c^The results of proliferation assays are expressed as the stimulation index (SI), calculated as the ratio between the mean counts per minute (cpm) for triplicate stimulated cultures and the mean counts per min for triplicate unstimulated cultures. SI values 2.5-fold greater than the SI of the control groups were considered as significant.

### Challenge study

**Figure 3:**
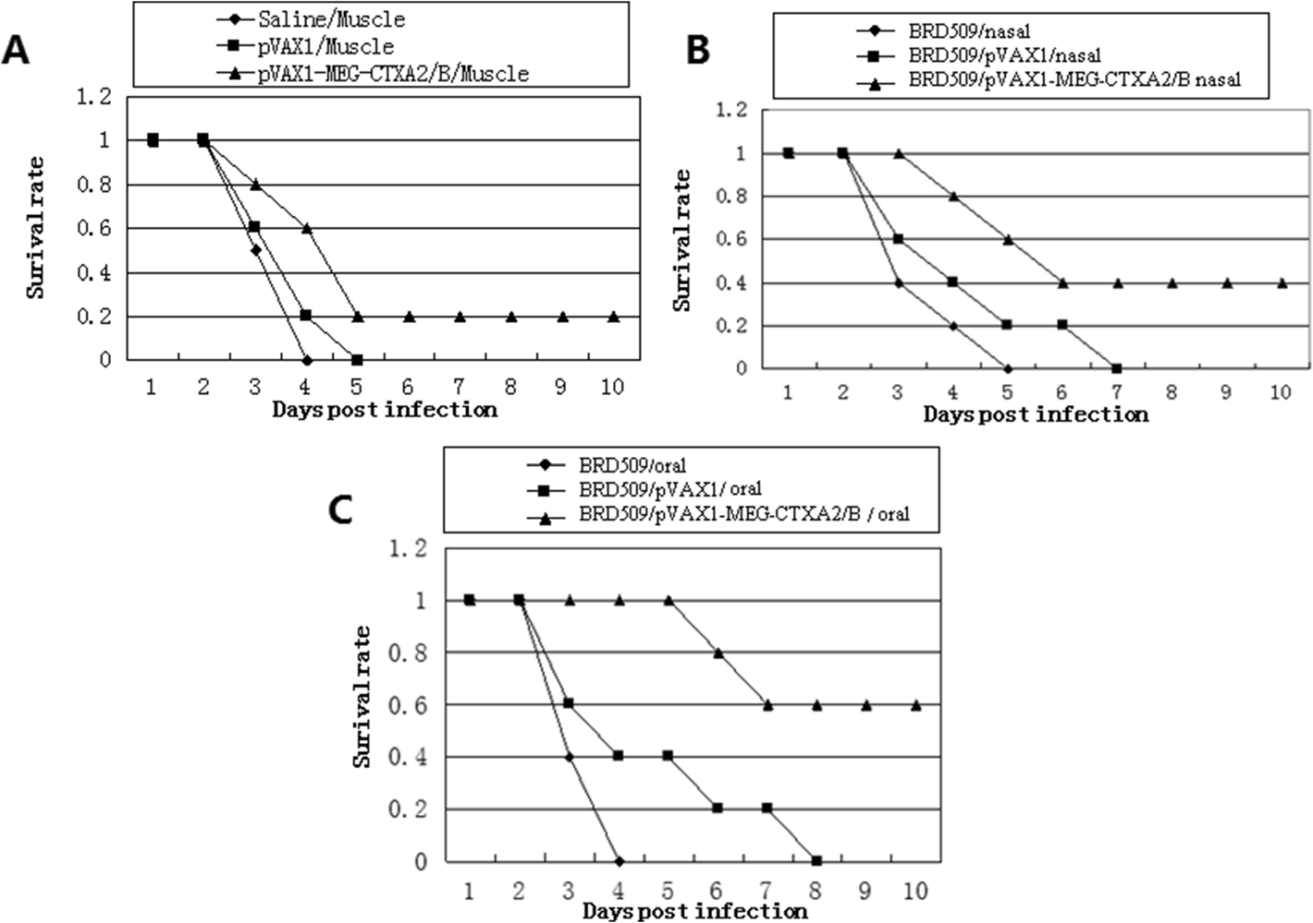
**Survival curves of immunized mice after challenge with 10**^**3 **^**tachyzoite forms of RH *T. gondii *strain 4 weeks after the last immunization. (A)** Intramuscular immunization group, the mice were injected with 100 μl 1 μg/μl recombinant plasmid, empty plasmid or PBS in the quadriceps muscle; **(B)** Nasal immunization group, each mouse was given a nasal drip of 50 μl 10^9^ cfu/ml *Salmonella* bacteria with recombinant plasmid, empty plasmid or empty bacteria; **(C)** Oral immunization group, the mice were administrated orally with 200 μl 10^9^ cfu/ml *Salmonella* bacteria with recombinant plasmid, empty plasmid, or empty bacteria.

Four weeks after the last immunization, mice were challenged with 100 ul 1 × 10^3^ tachyzoites of *T. gondii* RH strain intraperitoneally. All the control mice died. Mice treated with saline died within 4 days. Mice immunized with empty plasmid intramuscularly died within 5 days. Mice treated orally with empty *Salmonella* died within 8 days. While there was a 20% survival rate (2/10) for the mice immunized with pVAX1-MEG-CTXA_2_/B plasmid intramuscularly. The recombinant *Salmonella* BRD509/pVAX1-MEG-CTXA_2_/B intranasal immunization group had 40% survival rate (4/10) after 10 days. The highest survival rate of 60% (6/10) was achieved in the mice orally immunized with recombinant *Salmonella* BRD509/pVAX1-MEG-CTXA_2_/B (Figure 3).

## Discussion

Recently, considerable efforts have been devoted to the development of nucleic acid vaccine to protect against *T. gondii*[22-25]. However, because of the complexity of the parasite life cycle and the variability of the parasite antigens, multi-epitope vaccines have become an attractive strategy for development of vaccines against this parasite [26-28]. In this study, a DNA vaccine encoding seven epitopes of *T. gondii*, derived from the tachyzoite proteins SAG1, GRA1, ROP2, GRA4 and bradyzoite proteins SAG2C, SAG2X of *T. gondii,* were constructed and linked to A_2_/B subunit of *cholera toxin*.

However, DNA vaccine as a promising vaccine remains a challenge for an improved delivery system. Mucosal vaccines can enhance mucosal and systemic humoral immunity especially for intracellular parasites such as *T. gondii*[29,30]. Thus, the best way for a vaccine to induce mucosal immunity is directly by inoculation of the mucosal surface. In this study, attenuated strains of *S. typhimurium* BRD509 were used as vaccine vectors to deliver heterologous antigens to the mucosal surface of the vaccine recipients [31,32]. A recombinant attenuated *S. typhimurium* vaccine encoding a multi-epitope gene of *T. gondii* was constructed. The mice were given inoculations via oral, nasal, or muscular routes. Immune responses induced by oral or nasal immunization with attenuated recombinant *Salmonella* BRD509 vaccines were compared to immune response induced by immunization with the plasmid pVAX1-MEG-CTXA_2_/B DNA vaccine intramuscularly.

The results showed intramuscular immunization of mice induced higher levels of IgG antibodies than attenuated recombinant *Salmonella* vaccine immunization via oral and nasal routes. However, the attenuated recombinant *Salmonella* oral and intranasal immunization induced higher levels of IgA antibodies than intramuscular immunization. IgA is important in mucosal immunity to oral infection with toxoplasma cysts. Since antibodies of this isotype are important in preventing re-infection with *T. gondii*, inducing IgA may be a major strategic aspect of vaccine development [33,34].

Major mechanisms by which immunocompetent hosts control *T. gondii* infections is thought to be the strong and persistent cell-mediated immunity elicited by parasites [35,36]. In this study, splenocyte proliferation was enhanced in mice immunized by all three routes. Proliferation activity was significantly enhanced in mice immunized with recombinant attenuated *Salmonella* BRD509 via oral and nasal routes.

Both CD4^+^ and CD8^+^ T cell subsets play a central role in the establishment of protective immunity in hosts. In this study, CD4^+^ and CD8 ^+^ T lymphocytes subsets from immunized mice were assayed by flow cytometry. There was a marked increase in the percentage of CD8^+^ T-cells in mice immunized with the recombinant attenuated *Salmonella* BRD509 vaccine perorally. As we know, the memory T-cell consists of CD4^+^ and CD8^+^ T-cells, which can rapidly acquire effector functions to kill infected cells and secrete inflammatory cytokines that inhibit replication of the pathogen. Effector CD4^+^ T-cells enhance CD8^+^ T cells develope and help B-cell responses through the activation of antigen-presenting cells or the secretion of cytokines, such as IFN-γ, IL-2 [37,38]. Compared with the intramuscular immunization DNA vaccine, introduction of attenuated *Salmonella* further enhanced Th1 cell-mediated immunity with higher levels of IFN-γ and IL-2 but low levels of IL-4 and IL-5. These results clearly demonstrate that attenuated *Salmonella* BRD509 can significantly augment Th1-type cellular immune responses in BALB/c mice. Both T-cell subsets are important sources of IFN-γ. Optimal protective CD8^+^ T cell responses depend on the ability of CD4^+^ T-cells to provide the growth factor IL-2.

In this study, a highly virulent RH strain of *T. gondii* was used for challenge study. When challenged with lethal doses of *T. gondii* (1 × 10^3^) all three immunization groups prolonged survival time compared to control mice. Especially, mice immunized perorally had a 60% survival rate. There was somewhat less enhancement of survival (40%) following intranasal immunization, while only a 20% survival rate was achieved following intramuscular immunization. In the future, a cyst-forming PRU strain of *T. gondii* will be used to evaluate the protective potency of this recombinant *Salmonella* vaccine via the mucosal immunization route.

## Conclusion

In summary, multi-epitope vaccines delivered by attenuated *Salmonella* are a promising way to elicit protective immune responses. Compared to intramuscular injection, oral and nasal immunizations with recombinant *Salmonella* elicits both systemic and mucosal immune responses, accompanied by a significant increase in survival rates in vaccinated mice after challenge with highly virulent parasites. The results from this study should contribute to development of highly protective mucosal vaccines against *T. gondii*.

## Competing interests

The authors declare that they have no competing interests with this publication.

## Authors’ contributions

HC carried out the vaccine construction and drafted the manuscript. XZ performed the immune response analysis. XZ and LZ performed the animal experiments. QY and ZW participated in the design of the study. QY performed the statistical analysis. YZ and SH made a revision of the manuscript. All authors have read and approved the final manuscript.
